# Supplementary material for: Monitoring the health of wolves (Canis lupus): Integrating conservation and public health
Source: PLoS One. 2026 Jan 14;21(1):e0338969. doi: 10.1371/journal.pone.0338969 (PMC12834336; doi:10.1371/journal.pone.0338969)
Supplement: Attachment — Submitted filename: Response to Reviewers.docx [file pone.0338969.s002.docx]

**Response to Reviewers**

**Padova, 18^th^ November 2025**

**Manuscript title: Monitoring the Health of Wolves (*Canis lupus*): Integrating Conservation and Public Health**

**PONE-D-25-46208**

Dear Editor,

We thank the Reviewers for their valuable comments and constructive suggestions, which have helped us to improve the quality and clarity of our manuscript. Below, we provide a detailed response to each comment.

**Addendum 1 (13^th^ November 2025):** In order to fully comply with the *PLOS ONE* submission guidelines regarding copyright, we have modified Figures 1 and 3 by removing the copyrighted maps. The figure captions have been updated accordingly in this second resubmission.

Furthermore, regarding the identifying data in the Supporting Information file Table S1.xlsx, we carefully verified that no identifying information is present. The dataset does not contain any data related to humans; all data refer exclusively to fecal samples collected from wolves (and one dog) in the environment, with no direct contact with the animals.

**Addendum 2 (18^th^ November 2025):** As requested, we checked the copyright status of Figures 1 and 3. We confirm that the shapefile of the Park’s boundaries we used is freely available online. To ensure maximum transparency, we have now stated its use and source explicitly in the Materials and Methods section.

Furthermore, to clarify that the data on wolf packs derive from Dissegna et al. (2023), we have revised the captions of Figures 1 and 3 accordingly.

Finally, we have re-uploaded Figure 1 after adjusting the position of the legend, scale bar, and compass to improve the overall layout.

**Reviewer #1**

Dear Authors,

This is a very interesting study reporting data on some viruses and endoparasite infections of wolves in an Italian national park, by examining a fairly large number of wolf fecal samples collected over almost a year (between May 2019 and March 2020). Overall, the manuscript is extremely well conceived and written, and the results are really very interesting. However, the manuscript shows some small imperfections in the way the names of the studied pathogens were written, while the discussion on the sensitivity of the method used for the detection of Echinococcus granulosus and Echinococcus multilocularis eggs deserves further exploration. More detailed comments are reported below.

**Reply: We thank the Reviewer for comments and suggestions. The manuscript was revised accordingly, as specified below.**

R#1 Line 26: Sarcocystis spp. are not helminths

**Reply: We replaced helminths with a more appropriate term, that is “endoparasites”.**

R#1 Line 27: Please, replace Coccidia with coccidia

**Reply: We replaced coccidia as suggested.**

R#1 Line 38: Please, replace extirpated with a more appropriate term

**Reply: We changed with “driven to near extinction”.**

R#1 Lines 56 and 59: Carnivore Protoparvovirus 1, Canine Enteric Coronavirus and Canine Parvovirus 2 should not be written in italics

**Reply: We corrected the italics as requested.**

R#1 Lines 153-154: The coproscopic method used is not highly sensitive for the detection of Taeniid eggs. For this reason, other available methods are more frequently used, see Mathis A, Deplazes P, Eckert J. An improved test system for PCR-based specific detection of Echinococcus multilocularis eggs. Journal of Helminthology. 1996;70(3):219-222. doi:10.1017/ S0022149X00015443. This needs to be discussed.

**Reply: We thank the Reviewer for this valuable comment. We modified the M&M – *Parasitological analysis* section, the Results, and the Discussion to provide a clearer and more detailed description of our testing procedures.**

**Before performing the parasitological and virological analyses, we attempted to genotype all samples to identify the host species and individual wolves. As described in the manuscript, this process had a success rate of approximately 50%, and we successfully identified 81 unique genotypes (that means 81 individuals) from 132 samples (the dog sample was excluded). The other 51 samples successfully genotyped were second or third samples belonging to the same individual and therefore we selected the most recently collected sample for each identified individual.**

**Subsequently, we performed a real-time duplex PCR on all successfully genotyped samples (n = 80), regardless of their copromicroscopic results, as well as on all samples positive for taeniid eggs at copromicroscopy (n = 24), in an attempt to roughly assess the prevalence (percentage of positive among individuals; i.e., among genotyped samples) and to increase the probability to detect the parasite if circulating (including not genotyped samples). This approach followed the method proposed by Obber et al. (2022;** [**https://doi.org/10.1371/journal.pone.0268045**](https://doi.org/10.1371/journal.pone.0268045)**), who demonstrated that qPCR directly applied to feces is a recommended strategy and allows a reduction in the number of samples required to detect infection. Based on previous research in the same area (Poglayen et al., 2017;** [**http://dx.doi.org/10.1016/j.ijppaw.2017.01.001**](http://dx.doi.org/10.1016/j.ijppaw.2017.01.001)**), we expected a low prevalence of *Echinococcus* spp. (about 5%), and a sample size of around 100 was deemed sufficient to detect the parasite at an expected prevalence of about 5% or more.**

R#1 Line 155: Please, replace cysts with (oo)cysts

**Reply: We replaced cycts with (oo)cysts.**

R#1 Line 280: "Taeniidae is the most relevant parasitic genus"; Taeniidae is a parasitic family and not a parasitic genus

**Reply: We corrected the term as suggested.**

R#1 Lines 284-288: Having used a technique that is not highly sensitive for the detection of Taeniids, even the use of PCR can give false results if performed only on samples that tested positive to copromicroscopy

**Reply: As noted in our response above, we revised the text accordingly to enhance the clarity and transparency of our methodological description.**

R#1 Line 296: Please, replace taeniids with taeniid

**Reply: We replaced taeniids with taeniid as requested.**

R#1 Lines 280-359: In the discussion section, parasitological data should also be compared with those obtained in the following recent studies:

1. Cafiero SA, Petroni L, Natucci L, Tomassini O, Romig T, Wassermann M, Rossi C, Hauffe HC, Casulli A, Massolo A. New evidence from the northern Apennines, Italy, suggests a southward expansion of Echinococcus multilocularis range in Europe. Sci Rep. 2025 Mar 1;15(1):7353. doi: 10.1038/s41598-025-91596-7

2. Minichino A, Ciuca L, Dipineto L, Rinaldi L, Montagnaro S, Borrelli L, Fioretti A, De Luca Bossa LM, Garella G, Ferrara G. Exposure to selected pathogens in wild mammals from a rescue and rehabilitation center in southern Italy. One Health. 2025 Apr 21;20:101049. doi: 10.1016/j.onehlt.2025.101049

3. Cafiero SA, Petroni L, Natucci L, Casale L, Raffaelli M, Baldacci D, Di Rosso A, Rossi C, Casulli A, Massolo A, Hauffe HC, Perrucci S. Parasite diversity in grey wolves (Canis lupus) from Tuscany, central Italy: a copromicroscopical investigation. Int J Parasitol Parasites Wildl. 2025 May 31;27:101092. doi: 10.1016/j.ijppaw.2025.101092

**Reply: We thank the Reviewer for these suggestions. We have added references (1) and (3) in the Discussion section. Reference (2) was not included, as we consider its epidemiological relevance to be limited, given that only four wolves were screened.**

R#1 Lines 368-369: This sentence should be deleted or changed according to comments reported above

**Reply: We thank the Reviewer for this comment. We have added a sentence highlighting that, although copromicroscopy can be considered a valuable tool for monitoring, its low sensitivity toward certain groups of parasites (such as taeniids) should be taken into account when interpreting the results.**

R#1 Line 380: Please, replace Reference with References

**Reply: We corrected accordingly.**

R#1 Lines 381-553: In the references, parasite and animal genera and species should be written in italics

**Reply: Text was edited accordingly.**

**Reviewer #2:**

R#2 Line 43 - What type of legal protection?

**Reply: We added in text “Habitat Directive 92/43/EEC and Italian Law 157/1992” to clarify the legal protection.**

R#2 Lines 50-51 - Include an example of a zoonotic disease that corresponds to this assertion.

**Reply: We thank the Reviewer for this suggestion. We have added an example of a pathogen that may threaten wolf conservation, canine distemper virus, and one that is relevant to public health, for which wolves may act as spreaders, *Echinococcus granulosus*.**

R#2 Line 82 - need scientific name of red fox

**Reply: The scientific name was included.**

R#2 Line 88 - Echinococcus granulosus sense strict includes G1-G3 but just G1 is mentioned here, what about G2 and G3?

**Reply: We corrected the genotypes of *Echinococcus granulosus* sensu stricto adding “(G1-G3)”.**

R#2 Line 96 - wolf should be wolves

**Reply: We corrected with wolves.**

R#2 Line 158-159 - which unique genotypes are tested for?

**Reply: We thank the Reviewer for this comment that helped us to clarify the genetic analysis performed. We modified the description in the Materials and Methods – *Sample collection* section, specifying that genetic analyses were performed to determine the host species and individual identity of wolves. In addition, we revised the *Parasitological analysis* section to further clarify this aspect. The detailed analysis of wolf genotypes is beyond the scope of the present study, as it is fully reported in another article by Dissegna et al. (2023), as indicated in the manuscript.**

R#2 Line 287 - again which genotypes were test for?

**Reply: Following the comment above and the comments of Reviewer 1, we also modified this phrase: “Following the method proposed by Obber et al. (2022), to increase the probability of detecting *E. granulosus* and *E. multilocularis*, we performed a method with high sensitivity (i.e., a real-time duplex PCR) on all samples attributable to individual wolf genotypes (n=80) - regardless of their copromicroscopic results - as well as on the samples belonging to unidentified wolves that tested positive for taeniid eggs at copromicroscopy (n=24).”**

R#2 Generally an interesting and well written paper. Are Parvo and Coronavirus really the only two viruses of concern between feral/domestic dogs and wolves? Perhaps dive a little more into why these two were chosen and their history in the area, in contrast to other transmissible viruses of concern.

**Reply: We thank the reviewer for this valuable suggestion. We selected canine parvovirus (CPV) and canine Ccoronavirus (CCoV) as target pathogens because both have been repeatedly reported in Italian wolf populations, including previous CPV cases documented in the Foreste Casentinesi National Park. These viruses are highly contagious, environmentally stable, and can persist in fecal material, making them ideal candidates for detection through copromicroscopy. Additionally, CPV and CCoV are well-known examples of pathogens that circulate at the interface between domestic dogs and wild carnivores, representing effective indicators of dog–wolf interactions. Other viral agents such as canine distemper virus (CDV), adenoviruses, and rabies virus are also relevant; however, their detection typically requires blood or tissue samples, which were not available in our non-invasive sampling design. We added a phrase in the M&M – *virological analysis* section to clarify these aspects.**
